# Surface Modification of Polylactic Acid Bioscaffold Fabricated via 3D Printing for Craniofacial Bone Tissue Engineering

**DOI:** 10.3390/ijms242417410

**Published:** 2023-12-12

**Authors:** Yao-Chang Liu, Guan-Jie Lo, Victor Bong-Hang Shyu, Chia-Hsuan Tsai, Chih-Hao Chen, Chien-Tzung Chen

**Affiliations:** 1 Department of Plastic and Reconstructive Surgery, Keelung Chang Gung Memorial Hospital, Keelung 204, Taiwan; c5793@cgmh.org.tw (Y.-C.L.); mpq967@cgmh.org.tw (G.-J.L.); m3411@cgmh.org.tw (V.B.-H.S.); chtsai0715@cgmh.org.tw (C.-H.T.); 2 Division of Trauma Plastic Surgery, Department of Plastic and Reconstructive Surgery, Chang Gung Memorial Hospital at Linkou, Craniofacial Research Center at Taoyuan, College of Medicine, Chang Gung University, Taoyuan 333, Taiwan

**Keywords:** bone tissue engineering, three-dimensional printer, three-dimensional printing scaffolds, bone defect repair

## Abstract

Bone tissue engineering is a promising solution for advanced bone defect reconstruction after severe trauma. In bone tissue engineering, scaffolds in three-dimensional (3D) structures are crucial components for cell growth, migration, and infiltration. The three-dimensional printing technique is well suited to manufacturing scaffolds since it can fabricate scaffolds with highly complex designs under good internal structural control. In the current study, the 3D printing technique was utilized to produce polylactic acid (PLA) scaffolds. BMSCs were seeded onto selected scaffolds, either hydrogel-mixed or not, and cultivated in vitro to investigate the osteogenic potential in each group. After osteogenic incubation in vitro, BMSC-seeded scaffolds were implanted onto rat cranium defects, and bone regeneration was observed after 12 weeks. Our results demonstrated that BMSCs were able to seed onto 3D-printed PLA scaffolds under high-resolution observation. Real-time PCR analysis showed their osteogenic ability, which could be further improved after BMSCs were mixed with hydrogel. The in vivo study showed significantly increased bone regeneration when rats’ cranium defects were implanted with a hydrogel-mixed BMSC-seeded scaffold compared to the control and those without cell or hydrogel groups. This study showed that 3D-printed PLA scaffolds are a feasible option for BMSC cultivation and osteogenic differentiation. After mixing with hydrogel, BMSC-seeded 3D-printed scaffolds can facilitate bone regeneration.

## 1. Introduction

Reconstruction of craniofacial bone defects is challenging for surgeons. For now, autologous bone grafting is the most widely used procedure to restore bone defects. However, the need to harvest autologous bone grafts increases the risk of donor site morbidities, such as pain, infection, or nerve damage. Allogenic bone grafting is an alternative solution, but the risks of immunologically mediated rejection, disease transmission, or infection are major concerns of allograft failure [1,2,3]. To overcome the aforementioned disadvantages, bone tissue engineering provides a potential option to regenerate bone defects via an ex vivo cell culture, scaffolds, and growth factors [4,5,6].

A relevant selection of cells, biomaterial scaffold, and growth-stimulating signals are three crucial components for a successful tissue engineering construct—the so-called tissue engineering triad [7,8,9,10]. In bone tissue engineering, cells that possess osteogenic potential, like adipose-derived stem cells (ASCs) and bone-marrow-derived mesenchymal stem cells (BMSCs), are the most commonly used cell sources [11,12]. The advantages of ASCs include their accessibility and abundant resources for harvesting a large number of cells under minor invasive procedures [13,14]. Despite the multiple differentiation potential, the osteogenic differentiation efficacy of ASCs is less favorable compared to BMSCs [1,15]. BMSCs are harvested from bone marrow aspiration, but lower cell yields from the procedure require a longer time for cell expansion; meanwhile, the potential morbidities from the procedure impede its clinical application [16]. However, BMSCs share a closer differentiation lineage in osteogenesis with superior osteogenic ability and are, therefore, extensively used in bone tissue engineering [1]. A previous study demonstrated that a BMSC-seeded cryogel scaffold could effectively promote bone regeneration in rat cranium defect models after 12 weeks of implantation [17].

Since bone has a three-dimensional (3D) configuration, scaffolds with a 3D structure play an important role in bone tissue engineering, especially in complex 3D bony reconstruction. An ideal scaffold is composed of biocompatibility, biodegradability, and osteoconduction with proper mechanical properties so that it mimics the bone structure and acts as a temporary matrix for cell proliferation, extracellular matrix deposition, subsequent bone ingrowth, and eventually bone tissue regeneration [18,19,20]. Various scaffold fabrication techniques have been proposed for bone tissue engineering, including fiber bonding, phase separation, solvent casting, particulate leaching, membrane lamination, molding, and foaming [21,22]. However, these techniques have the major disadvantage that they do not have enough control over scaffold architecture, pore size, and pore network, which generates inconsistent 3D scaffolds and results in less favorable cell migration and infiltration [23,24].

To overcome the aforementioned problems in previous scaffold fabrication techniques, rapid prototyping techniques, and especially biocompatible 3D printing, are promising and innovative options. Three-dimensional printing techniques are computerized fabrication techniques that can produce complex 3D objects using data generated by computer-assisted design (CAD) systems [20,25,26,27]. This technique employs inkjet technology to process powder materials and print materials layer by layer to build up the full structure of desired objects during fabrication [19]. Due to its capability to fabricate customized scaffolds with controlled pore size and pore structure, it is well suited to generate scaffolds for bone tissue engineering [20,23]. Various degradable polymers, including poly-acrylonitrile butadiene styrene (ABS), polycaprolactone (PCL), polylactic acid (PLA), polyglycolic acid (PGA), and chitosan, can be used to fabricate 3D scaffolds [28,29,30,31,32,33]. Among these materials, PLA is a thermoplastic and biodegradable aliphatic polyester derived from a naturally organic acid and possesses excellent mechanical properties, making it an ideal material in bone tissue engineering [34,35,36].

Though PLA material provides desirable and tunable properties to manufacture customized scaffolds for tissue engineering, there are still limitations regarding cell–surface interaction because of its post-fabrication surface, hydrophobicity, and lack of recognizable biochemical binding sites [21,37,38]. Several surface modification techniques are developed to reduce barriers to cell adhesion, proliferation, and differentiation, such as topographical modification, protein adsorption, mineral coating, functional group incorporation, and biomacromolecule immobilization [37,39]. Meanwhile, with the advancement of biomaterial characteristics in hydrogels, they provide opportunities for more finely tuned characterization of cell responses to properties [40,41].

Despite the wide application of the 3D printing technique in bone tissue regeneration, the most optimal scaffold design, material, and cell-surface modification are still under investigation. Accordingly, this study aims to utilize the 3D printing technique to produce biodegradable scaffolds for bone tissue engineering, and hydrogel was applied to construct a bioactive environment. The osteogenic ability and bone regeneration potential of BMSCs seeded with 3D-printed scaffolds were investigated via in vitro cultivation and an in vivo rat cranium defect model.

## 2. Results

### 2.1. Three-Dimensionally Printed PLA Scaffold

**Figure 1:**
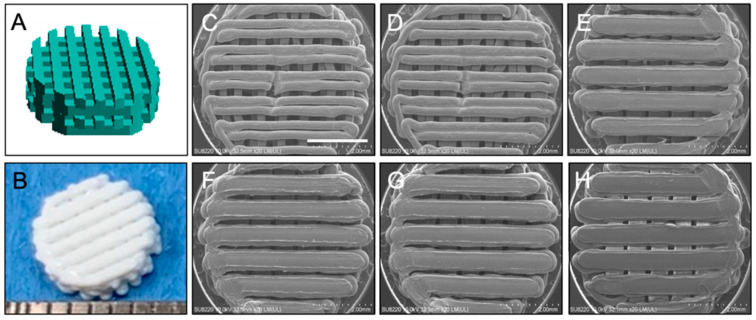
(**A**) The design of PLA scaffold using SolidWorks software, version 27. (**B**) Gross view of PLA scaffold and under SEM with infill rate (**C**) 64%; (**D**) 66%; (**E**) 68%; (**F**) 70%; (**G**) 80%; and (**H**) 100%. Scale bar = 2 mm.

The PLA scaffold was designed using SolidWorks software, version 27 (Dassault Systèmes SA, Vélizy-Villacoublay, France) as shown in Figure 1. During the process of scaffold printing, we found that different input infill densities produced scaffolds with different porosities. These 3D-printed scaffolds were observed under SEM to determine filament width and pore size. Results showed that as infill density was lowered, the filament width of the printed scaffolds decreased while the pore size increased. When infill density was set to 100%, 80%, 70%, 68%, 66%, and 64%, the corresponding filament width and pore size were 0.679 mm and 0.125 mm; 0.690 mm and 0.133 mm; 0.689 mm and 0.128 mm; 0.645 mm and 0.165 mm; 0.637 mm and 0.181 mm; and 0.591 mm and 0.214 mm. If infill density was below 64%, the 3D printer would not be able to print intact PLA scaffolds with acceptable porosity.

Printed PLA scaffolds underwent a degradation test in distilled water and were retrieved on weeks 2, 4, and 8. Weights were recorded and weight loss percentages were calculated. The measured weight slightly decreased during the test, and the calculated weight loss percentage only slightly increased. These scaffolds were implanted into the subcutaneous layer of nude rats’ dorsal skin and retrieved at 2, 4, and 8 weeks for in vivo degradation profiles. Likewise, there were no significant changes noted in scaffold structure before and after implantation.

### 2.2. Surface Modification of PLA Scaffolds by Hydrogel

**Figure 2:**
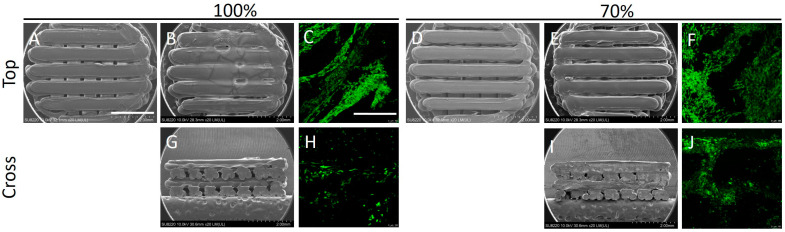
Surface modification of PLA scaffolds by hydrogel. (**A**) Top view of PLA scaffold under SEM observation with 100% infill rate. (**B**,**G**) Top and cross-section views of PLA scaffold modified with hydrogel. (**C**,**H**) Top and cross-section views of Live/Dead staining. (**D**) Top view of PLA scaffold under SEM observation with 70% infill rate. (**E**,**I**) Top and cross-section views of PLA scaffold modified with hydrogel. (**F**,**J**) Top and cross-section views of Live/Dead staining. Scale bar = 2 mm.

A cell suspension containing BMSCs was mixed with hydrogel and gently pipetted onto PLA scaffold with infill rates of 70% and 100% and observed under SEM as shown in Figure 2. Under SEM magnification, hydrogel infiltrated into the PLA scaffold and distributed more evenly when the scaffold was printed at a 70% infill rate. Meanwhile, higher cell density was noted in Live/Dead staining after 4 days of incubation at a 70% infill rate for the BMSC-seeded PLA scaffold. These findings implied better surface modification when utilizing hydrogel in scaffolds printed at a 70% infill rate.

### 2.3. BMSCs Viability in PLA Scaffolds

**Figure 3:**
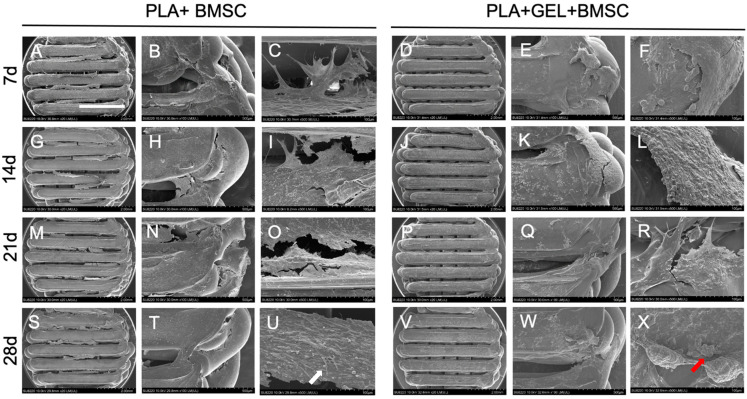
SEM of BMSC-seeded PLA scaffold with hydrogel-mixed or not on days 7, 14, 21, and 28. (**A**,**G**,**M**,**S**,**D**,**J**,**P**,**V**) Scaffold under 20-fold magnification. (**B**,**H**,**N**,**T**,**E**,**K**,**Q**,**W**) Scaffold edge under 100-fold magnification. (**C**,**I**,**O**,**U**,**F**,**L**,**R**,**X**) Inter-filament space within scaffold and scaffold edge under 500-fold magnification. White arrow, spindle-shaped cell. Red arrow, granular-shaped cell. Scale bar = 2 mm.

BMSCs with or without hydrogel were seeded onto PLA scaffolds printed at an infill rate of 70% and were cultured in osteo-inductive media. These scaffolds were observed under SEM on days 7, 14, 21, and 28 (Figure 3). Under SEM 500-fold magnification, BMSCs were able to seed onto PLA scaffolds evenly in the BMSC-only group. Spindle-shaped BMSCs adhered to scaffold surface and edges with cell colonies found within pores. Cell density was found to increase with time. These findings demonstrated the ability of BMSCs to grow on a PLA scaffold. In the other group, BMSCs mixed with hydrogel were also distributed onto the scaffold surface and edges evenly, and spindle-shaped cells could be found at these sites by day 7. However, cells within scaffold pores did not possess the spindle shape and remained in a granular shape until day 21.

**Figure 4:**
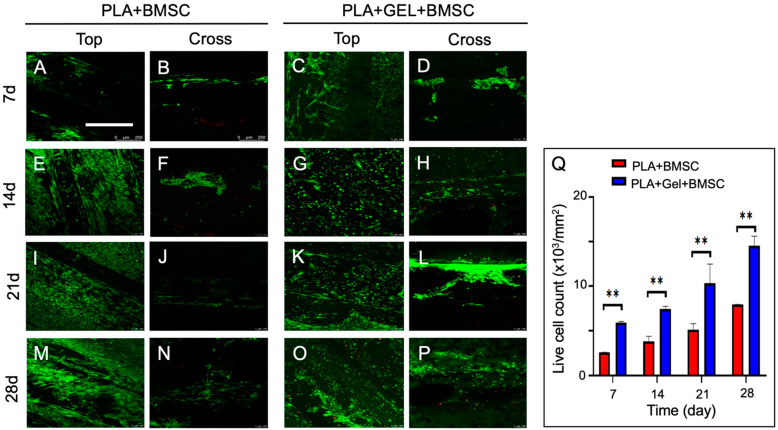
Live/Dead cells of BMSC-seeded scaffold under 10-fold magnification fluorescence microscopy on days 7, 14, 21, and 28. (**A**,**E**,**I**,**M**,**B**,**F**,**J**,**N**) Top and cross view of scaffold cells viability. (**C**,**G**,**K**,**O**,**D**,**H**,**L**,**P**) Top and cross view of hydrogel-mixed scaffold cells viability. (**Q**) Live cell counts between two groups during incubation. *p* < 0.01 **.

Cell-seeded PLA scaffolds incubated under osteo-inductive conditions were observed with confocal fluorescence microscopy after Live/Dead assay staining on days 7, 14, 21, and 28. Cell viability could be interpreted according to fluorescence color under microscopy, where red represented dead cells and green represented live cells (Figure 4). Cells adhered to scaffold surfaces with little dead cells noted, which implies that PLA scaffold can be used for cell culture without endangering cell growth. PLA scaffolds were cut in a sagittal section and observed under microscopy. Cells proliferated in the sagittal plane during incubation showing that the porosity of scaffolds allowed cells to migrate inward. Meanwhile, cell-seeded scaffold mixed with hydrogel demonstrated better inward growth ability. ImageJ software, version 1.54f (National Institute of Health, Bethesda, MD, USA) was used to analyze Live/Dead cell counts and hydrogel-mixed BMSC-seeded scaffold had a higher live cell count during the incubation period.

### 2.4. Osteogenic Potential of BMSCs Cultured in PLA Scaffolds

**Figure 5:**
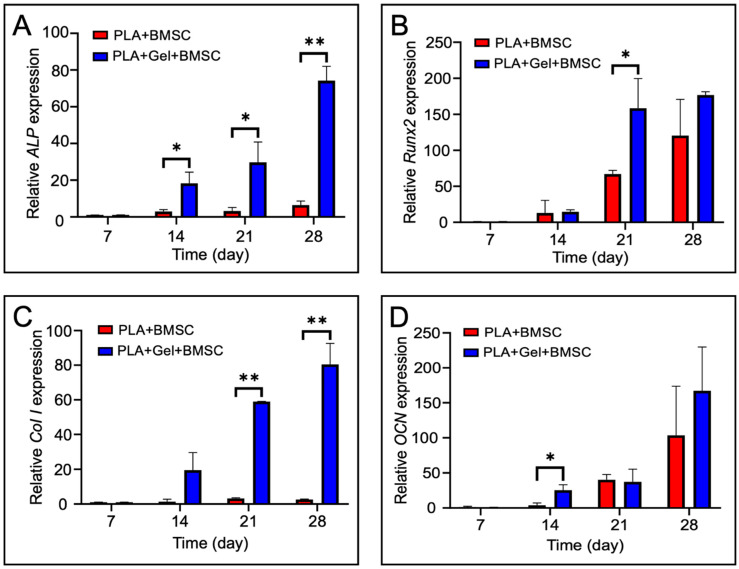
Osteogenic differentiation genes expression between BMSC-seeded PLA scaffold (PLA + BMSC) and hydrogel-mixed BMSC-seeded PLA scaffold (PLA + GEL + BMSC). (**A**) *ALP* expression. (**B**) *Runx2* expression. (**C**) *Col 1* expression. (**D**) *OCN* expression. *p* < 0.05 *, *p* < 0.01 **.

Quantitative real-time PCR analysis was carried out after 7, 14, 21, and 28 days of culturing to evaluate the expression of osteocalcin (*OCN*), alkaline phosphatase (*ALP*), collagen type I (*Col 1*), and *Runx-2*. In the BMSC-seeded PLA scaffold (PLA + BMSC) group, expression of the above genes increased during incubation, and the level of gene expression was higher in hydrogel-mixed BMSC-seeded PLA scaffold (PLA + GEL + BMSC) group. (Figure 5).

### 2.5. In Vivo Bone Regeneration Study

**Figure 6:**
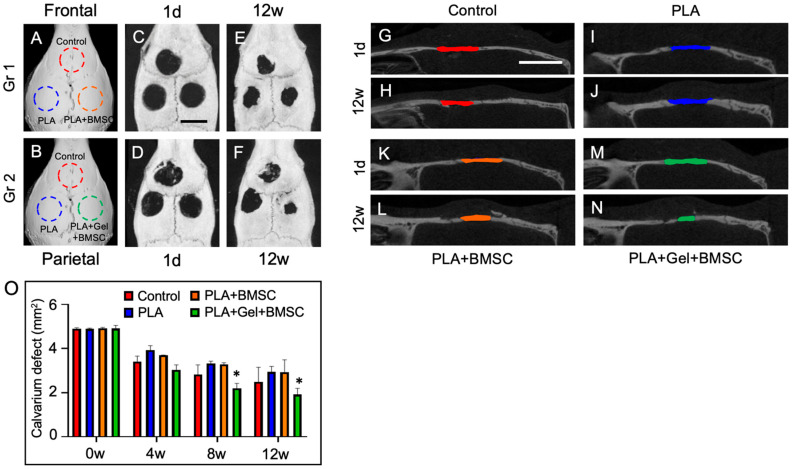
Bone regeneration examined by Micro-CT. (**A**–**N**) Cranium defects with different implantation in experimental rats and Micro-CT image at post-implantation day 1 and week 12. (**O**) Defect size was measured using ITK-SNAP software, version 4.0.2 (Penn Image Computing and Science Laboratory (PICSL), Philadelphia, PA, USA), *p* < 0.05 * and the colored bars in each image represented remaining defect size. (Red = Control; Blue = PLA; Orange = PLA +BMSC; Green = PLA + Gel + BMSC) Side bar = 4 mm.

After 7 days of culture in osteo-inductive media, both groups of BMSC-seeded PLA scaffolds were implanted into rat cranium defects. Bone regeneration was examined by micro-CT on day 1 and at weeks 4, 8, and 12 after implantation procedures (Figure 6). After 12 weeks of follow-up, micro-CT showed bone regeneration in cranium defects. More bone regeneration was noted in defects implanted with hydrogel-mixed BMSC-seeded PLA scaffolds (PLA + GEL + BMSC) than those implanted with BMSC-seeded PLA scaffolds (PLA + BMSC). The defect volume was measured in each group. At post-implantation day 1, the volume of defects without implantations (defect) was 4.9 mm^2^; for those implanted with PLA scaffold (PLA), it was 4.98 mm^2^; for those with BMSC-seeded PLA scaffold (PLA + BMSC), it was 4.91 mm^2^; for those with hydrogel-mixed BMSC-seeded PLA scaffold (PLA + GEL + BMSC), it was 4.91 mm^2^. After 12 weeks, the defect volume of each group was as follows: defect—2.49 mm^2^; PLA—2.94 mm^2^; PLA + BMSC—2.93 mm^2^; PLA + GEL + BMSC—1.92 mm^2^.

Cranium defect volume was calculated using the ITK-SNAP software, version 4.0.2 (Penn Image Computing and Science Laboratory (PICSL), Philadelphia, PA, USA). The defect volume in the PLA + GEL + BMSC group was less when compared to other groups. PLA group defect volume was larger than other groups at post-implantation week 4, but its volume was close to the PLA + BMSC group by week 12. The defect volume in the defect group was larger than PLA + GEL + BMSC group but less than PLA and PLA + BMSC groups. The regeneration rate in each group was calculated with the below formula:

Regeneration rate (%) = [volume (1 day) − volume (12 week)]/volume (1 day) × 100%

The calculated regeneration rate in the PLA + GEL + BMSC group was the best among the groups, followed by the defect-only group. Regeneration rates in PLA and PLA + BMSC groups were similar.

**Figure 7:**
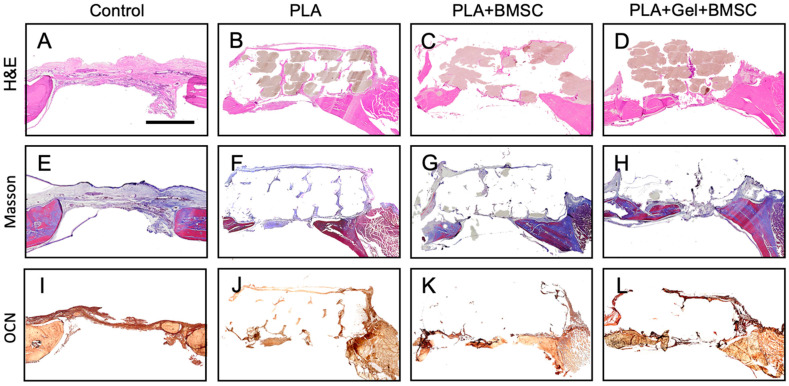
H&E, Masson Trichrome stain, and IHC stain of OCN of cranium defect in each group after 12 weeks of in vivo study. (**A**–**D**) H&E. (**E**–**H**) Masson. (**I**–**L**) OCN.

The cranium defects of interest were retrieved for cross-section after 12 weeks of in vivo implantation study. Each specimen was processed with hematoxylin and eosin (H&E) and Masson’s Trichrome stain for histology study. Histologically, the area of Masson’s Trichrome stain that matched with the H&E stain represented the bone regeneration status in each group (Figure 7).

## 3. Discussion

A 3D-printed PLA scaffold is a feasible solution in terms of BMSC cultivation and application in bone regeneration. The rat cranium defect implanted with a hydrogel-mixed BMSC-seeded PLA scaffold shows increased bone regeneration after 12 weeks of in vivo incubation. The application of hydrogel can facilitate BMSCs cultivation on 3DP scaffolds and thus improve bone regeneration when implanting scaffolds in vivo.

PLA is a thermoplastic and biodegradable polymer commonly used as a 3D printing material. Its strong biomechanical properties make it ideal for bone tissue engineering [34,35,36]. In our study, we used PLA as our 3D printing biomaterial and found that different infill densities would produce scaffolds with different porosity. The 3D printer could not successfully print an intact scaffold if the infill density was set below 64%. We chose a scaffold printed at 70% infill density and produced a scaffold with a filament width of 0.689 mm and pore size of 0.128 mm as our in vivo study scaffold. An in vitro hydrolytic degradation test showed a limited change in scaffold appearance and scaffold with a slight weight loss. In a previous study, the PLA material showed water absorbability which indirectly determined its hydrolytic degradation. However, its water absorption slightly increased when immersed in distilled water as a function of time, and the weight loss rate was less than 1% after the hydrolytic degradation test [42]. These findings suggest the biodegradability of the PLA scaffold, but that the degradation rate is relatively slow.

BMSCs are multipotential cells with powerful replicating capacity and have been reported to differentiate into bone, endothelium, adipose tissue, cartilage, muscle, and the brain [16,43,44]. Among these differentiation potentials, BMSC has a closer differentiation lineage for osteogenesis [1]. Immunologically, BMSC is able to modulate immune reactions in vitro and escape from immune surveillance in vivo in previous studies [45,46]. These properties make it an ideal source for tissue regeneration, especially in the field of bone tissue engineering. In this study, BMSCs were extracted from rats’ femurs and seeded onto printed PLA scaffolds. Under SEM observations, spindle-shaped BMSCs were distributed and grown on scaffolds evenly. Though granular-shaped cells were observed in hydrogel-mixed BMSC-seeded PLA scaffolds, live cell amount still increased and inward growth along scaffold pores was seen as a function of time in both groups. These findings suggest that 3D-printed PLA scaffolds are biocompatible for BMSC adhesion and growth, and hydrogel does not endanger cell expansion. The granular cells observed under SEM could be the result of cells wrapped in hydrogel.

The osteogenic differentiation of BMSCs includes three phases: proliferation, matrix maturation, and mineralization [47]. During the early stages of osteogenic differentiation, Runx2 is regarded as one of the most important transcription factors and has become a marker of early osteogenic differentiation. Once Runx2 is activated, cells are regarded as pre-osteoblasts and enter the cell proliferation phase. As osteoblast maturation progresses, these cells exit the cell cycle and begin to differentiate while maturing extracellular matrix with alkaline phosphatase (ALP) and the collagen type I alpha I chain (COL1A1). During the mineralization phase, cells gradually differentiate into mature osteoblasts as enriched osteocalcin (OCN) promotes mineral substance deposition [48,49,50,51]. In this study, we used a real-time PCR technique to detect gene expression after seeding BMSCs onto the PLA scaffold. The results showed that the level of *RUNX-2*, *ALP*, *COL1A1*, and *OCN* expression increases over the duration of osteo-inductive culturing, and the hydrogel-mixed BMSC-seeded scaffold showed a significantly increased level of osteogenic gene expression among the experimental groups.

Cell-seeded PLA scaffolds were implanted onto surgically created cranium defects of rats. Micro-CT and cross-sectioning were conducted to evaluate bone regeneration after 12 weeks of in vivo implantation. The regeneration rate of defects implanted with the hydrogel-mixed BMSC-seeded PLA scaffold was 60%, and the rate of defects implanted with BMSC-seeded scaffold was only 40.4%, similar to defects implanted with scaffold-only PLA, and lower than the control group. Previous studies have reported good tolerance of PLA materials to mesenchymal stem cells and the ability to promote cell adhesion onto scaffolds cultured in vitro with osteogenic differentiation potential [52,53]. These results are consistent with this study. However, when implanting PLA scaffolds onto rat cranium defects, groups implanted with scaffold-only PLA or BMSC-seeded PLA scaffold did not see accelerated bone regeneration in our animal model. Diomede et al. reported that a 3D-printed PLA scaffold showed no cytotoxicity but did not exhibit osteogenic inductivity after being implanted in rats with cortical calvaria bone defects after 6 weeks of in vivo study [54]. Han et al. also demonstrated that PLA scaffolds showed good biocompatibility but poor osteogenic capacity when implanting scaffolds into rats with tibia bone defects [55].

Several disadvantages of PLA material have been reported regarding its application in tissue engineering, including the slow degradation rate and the release of acidic degradation products [56,57]. As an ideal scaffold for bone tissue engineering should be characterized by a balanced resorption rate to provide space for newly forming bone tissue, [52] slow degradation of the material may occupy the space for tissue regeneration. Meanwhile, the release and accumulation of degradation acidic products from the PLA scaffold may decrease the surrounding tissue pH level and cause an inflammatory response, affecting cell growth and tissue regeneration [57,58]. To improve osteogenic capacity, several studies have been conducted to investigate better modification of PLA scaffold [59,60]. In this study, the hydrogel-mixed BMSC-seeded PLA scaffold increased bone regeneration after 12 weeks of implantation. Hydrogels have been widely used in the field of tissue engineering for their ability to replicate extracellular matrix (ECM)-like properties, providing a hydrated and mechanically stable environment while encapsulating cells to improve better adhesion or proliferation [61,62]. Improved cell proliferation, osteogenic differentiation, and bone regeneration were noted in the present study after mixing hydrogel with BMSC-seeded scaffolds, indicating a benefit from the properties of hydrogels.

The 3D printing technique offers an effective way to fabricate scaffolds for tissue engineering, and these 3DP scaffolds are one of the most promising approaches for hard-tissue regeneration and repair [63,64,65]. PLA provides desirable characteristics for tissue engineering, but there are limitations stemming from their poor surface properties, bulk properties, hydrophobicity, and lack of recognizable biochemical binding sites, which are necessary for cell–surface interaction [37,63]. To overcome these problems, many efforts have been directed at modifying the surface of the scaffolds, and hydrogels with advanced biomaterial characteristics are one of the solutions [37]. Bai et al. injected supramolecular hydrogels into the inner pores of a 3D printing porous metal scaffold and results showed significant change in the morphology of BMSCs, subsequent osteogenic differentiation, and cell proliferation [66]. Previous studies have shown that a PLA scaffold coated with gelatin could enhance its physicochemical properties and favor osteoblast differentiation [67,68]. In this study, we mixed a hydrogel with BMSCs and seeded them onto a 3D-printed PLA scaffold. Under SEM observation, the hydrogel was able to distribute evenly within scaffold pores, and subsequent results also showed better BMSC osteogenic differentiation, cell proliferation, and bone regeneration in vivo and in vitro, which implies the possible benefit of using a hydrogel to physically modify the scaffold surface, creating a bioactive environment and promoting bone regeneration.

There are limitations to our study. We aimed to verify the outcome of bone regeneration using 3D-printed scaffolds in BMSCs osteogenic differentiation. Though an in vivo study showed that bone regeneration improved after implantation of hydrogel-mixed BMSC-seeded PLA scaffolds, when PLA scaffolds or BMSC-seeded PLA scaffolds were implanted, bone regeneration was even slower than the control group, which implies possible interference in bone regeneration process. In hydrogel-mixed BMSC-seeded scaffolds, better osteogenic differentiation and bone regeneration were observed. Under SEM observation, the hydrogel was able to distribute within the porous structure of the PLA scaffold. We postulated a possible surface modification process between the hydrogel and PLA scaffold. However, we were not able to prove this viewpoint from the current study, and further comprehensive studies are warranted to investigate the optimal material and environment to satisfy bone regeneration for a BMSC-seeded scaffold implanted in vivo.

## 4. Materials and Methods

### 4.1. BMSCs Extraction and Expansion from Rats

BMSCs were extracted from 6-to-8-week-old Lewis rats and cultured. In brief, femurs were carefully harvested from experimental rats under anesthesia. Sterile low glucose Dulbecco’s Modified Eagle Medium (DMEM) was irrigated into the bone marrow cavity 2–3 times to fully flush out bone marrow cells. Bone marrow cells were then transferred onto a 15 cm Petri dish evenly, followed by culturing within a carbon dioxide incubator (5% CO_2_, 37 °C). The culture medium was replaced every 3 to 4 days. BMSCs gradually formed cell colonies after 7–10 days.

### 4.2. PLA Scaffold Preparation

Polylactic acid (3DXTech PLA, BDXTECH) filament was employed as the base material for 3D printing. PLA scaffold was designed in a cylinder shape and 4-layer vertical stacking fashions using SolidWorks software, version 27 (Dassault Systèmes SA, Vélizy-Villacoublay, France) with a diameter of 4 mm, height of 1.6 mm and infill line distance of 0.4 mm to create a uniform square porous structure. The diameter of the printed filaments was 400 μm, with a filament spacing of 410 μm. Subsequently, NewCreator K software, V1.57.49 (ROKIT Healthcare, Seoul, Korea) was used for further slicing, converting the design into a gcode file. The manufacturing of the 3D-printed scaffold was accomplished through the process of Fused Deposition Modeling (FDM), employing the ROKIT INVIVO Premium hot-melt air-extrusion 3D printer. The scaffold was constructed layer by layer along a predetermined path. The nozzle’s movement speed was approximately 5 mm/s, and the extrusion temperature of the 3D printer’s nozzle was set at 190 °C. The height of each cross-section of the scaffold was 0.4 mm, and the filaments between printing layers were oriented at a 90° angle. The scaffold consisted of 4 layers. Scaffolds were printed in different infill densities and each structure was examined under a scanning electron microscope (SEM) to determine appropriate settings. Meanwhile, BMSCs were seeded onto printed scaffold and live and dead analysis was conducted to determine the optimal scaffold. Eventually, experimental PLA scaffolds were printed in 70% infill density with a filament width of 0.689 mm and a pore size of 0.128 mm.

### 4.3. In Vitro Test

#### 4.3.1. Cell Cultures

BMSCs isolated from Lewis rats were divided into two groups for experimentation. In the first group, a cell suspension containing 7.5 × 10^5^ BMSCs was carefully pipetted onto printed PLA scaffolds. Conversely, in the second group, a cell suspension with the same cell concentration was mixed with a hydrogel composed of 4 mL GelMA (gelatin modified with methacryloyl groups) and 1 mL Gel-linker (GEL, Rokit Healthcare, Seoul, Korea). The cell suspension was evenly mixed with 4 mL GelMA, followed by the addition of 1 mL Gel-linker before being gently pipetted onto the printed PLA scaffolds prior to hydrogel crosslinking. Subsequent to cell seeding, BMSCs were cultured in low glucose DMEM for 4 days, followed by a 28-day period of osteo-inductive culturing. Sampling for further observations was conducted on days 7, 14, 21, and 28.

#### 4.3.2. Scaffold Degradation Test

Dry weight of prepared scaffolds (Wi) was measured and then incubated into 37 °C 3 mL distilled water. Scaffolds in each group were retrieved at weeks 2, 4, and 8, and dried at 100 °C. Weights of scaffolds after the degradation test (W0) were measured 24 h after drying. Weight loss percentage was calculated as:

Weight loss (%) = ((Wi − W0)/W0) × 100

#### 4.3.3. SEM Observation

Each group of cell-seeded scaffolds was immersed in 2.5% glutaraldehyde for 3 h at 37 °C, and washed with PBS 3 times for 10 min. Washed scaffolds were fixed with 1% ostium tetroxide (OsO_4_) for 2.5 h at 37 °C. After thoroughly washing with d2H2O, the samples were dehydrated in ethanol in a sequential manner (50%, 70%, 80%, 90%, 95% and 100%). After drying in liquid CO_2_, these samples were observed by SEM after gold coating.

#### 4.3.4. Live and Dead Test

Cell viability of cell-seeded scaffolds was verified using a Live/Dead cell viability assay kit (Sigma, St. Louis, MO, USA) under the manufacturer’s protocols. The Live/Dead staining solution was prepared with 1 µL reagent A (staining dead cells and emitting red fluorescence) and 2 µL reagent B (staining viable cells and emitting green fluorescence) in 1 mL PBS. After 30 min of reaction with the staining solution and thoroughly washing with PBS, the samples were observed and imaged under a fluorescence microscope for cell viability.

#### 4.3.5. Real-Time PCR

Cell-seeded PLA scaffolds were evaluated using real-time PCR on days 7, 14, 21, and 28 during osteo-inductive culturing. Total RNA of each specimen was isolated with Trizol (Invitrogen, Waltham, MA, USA) under the manufacturer’s instructions. RNA was treated with a Reverse First-Strand cDNA Synthesis kit (Fermentas, Waltham, MA, USA), and real-time PCR was carried out using an EZtime Real-Time PCR premix kit (Yeastern biotech, New Taipei City, Taiwan) with 1 μL of cDNA for 30 cycles. The primers used for osteogenic differentiation included collagen type I, osteocalcin, alkaline phosphatase, and Runx-2. All experimental steps were repeated 3 times, and glyceraldehyde-3-phosphate dehydrogenase (GAPDH) was used as an endogenous standard to normalize for input load of cDNA between the samples

### 4.4. In Vivo Study of Bone Regeneration

#### 4.4.1. In Vivo Animal Study

Lewis rats were used for an in vivo animal study. In total, 10 rats were used and separated into two groups (*n* = 5). Three 4 mm diameter cranium defects were created in each rat under anesthesia with standard operative procedures. In the first group, their first cranium defects were left without any material transplanted; the second defects were transplanted with printed PLA scaffold only; and the third defects were transplanted with BMSC-seeded PLA scaffolds. In the second group, the first and second cranium defects were managed the same as the first group, but their third cranium defects were transplanted with the BMSC-seeded PLA scaffold mixed with hydrogel (4 mL GelMA (gelatin modified with methacryloyl groups) + 1 mL Gel-linker) (GEL, Rokit Healthcare).

#### 4.4.2. Micro-CT

To assess calvarial bone regeneration of experimental rats, micro-computed tomography (CT) and imaging was carried out at week 4, 8, and 12 after implantation procedures. The volume of bone regeneration was measured by OsiriX, version 8.0.1 (Pixmeo SARL, Bernex, Switzerland) and ITK-SNAP software, version 4.0.2 (Penn Image Computing and Science Laboratory (PICSL), Philadelphia, PA, USA).

#### 4.4.3. Histological Examinations

The region of interest was dissected carefully until surrounding soft tissue was totally removed at weeks 4, 8, and 12 after implantation procedures. Each tissue specimen was decalcified with a decalcifying solution, followed by being embedded into paraffin and cut into sections. Histology included hematoxylin and eosin (H&E) and Masson’s Trichrome stain.

### 4.5. Statistical Evaluation

All data are presented as mean ± standard deviation (sd). Two-tailed nonparametric Kruskal–Wallis tests and Dunn’s multiple comparison post hoc test were performed among multiple groups using SPSS software, version 29 (SPSS Inc., Chicago, IL, USA). Statistical significance is considered as *p*-value < 0.05.

## 5. Conclusions

In the presented study, we utilized the 3D printing technique to produce PLA scaffolds, and BMSCs were seeded onto selected scaffolds, either hydrogel-mixed or not, and cultivated in vitro to investigate their osteogenic potential. After osteogenic incubation in vitro, BMSC-seeded scaffolds were implanted onto rat cranium defects, and observed bone regeneration over a 12-week period. Our results demonstrated that BMSCs were able to seed onto 3D-printed PLA scaffolds under high-resolution observation and real-time PCR analysis showed their osteogenic ability, which could be improved after mixing with hydrogel. An in vivo study showed significantly increased bone regeneration when rat cranium defects were implanted with hydrogel-mixed BMSC-seeded scaffold. This study highlights that a 3D-printed PLA scaffold is a feasible option for BMSC cultivation and osteogenic differentiation. After mixing with hydrogel, a BMSC-seeded 3D-printed scaffold can facilitate bone regeneration. Further studies are warranted to investigate the most optimal material and scaffold structure designs for bone regeneration.

## Data Availability

The data that support the findings of this study are available from the corresponding author upon an email request.
